# Climate Variability, Communal Violence, and Population Health in Africa’s Arc of Instability: A Scoping Review of Evidence and Gaps

**DOI:** 10.5334/aogh.5352

**Published:** 2026-07-03

**Authors:** Daniel Chigudu

**Affiliations:** 1 University of South Africa, Pretoria, South Africa

**Keywords:** climate variability, communal violence, population health, Sahel, Horn of Africa, scoping review, health systems, fragile states

## Abstract

*Background:* Africa’s arc of instability – a band of countries stretching from Mauritania through the Sahel to the Horn of Africa – experiences a convergence of climate variability, communal violence, and fragile health systems. Evidence on their joint operation remains fragmented across disciplines, limiting policy-relevant synthesis.

*Objectives:* This scoping review maps the published and grey literature on the joint operation of climate variability, communal violence, and population health in the arc of instability between January 2010 and March 2025; it identifies dominant pathways, populations, and methods, and articulates research gaps.

*Methods:* Following Arksey and O’Malley’s framework with Levac et al.’s refinements and reporting against the PRISMA-ScR checklist, five electronic databases (PubMed, Scopus, Web of Science, CINAHL, and Africa Wide Information) and grey literature from UN agencies, humanitarian organisations, and conflict and vulnerability databases were searched. Studies addressing at least two of the three domains in the arc, published in English or French, were included and synthesised narratively.

*Findings:* Of 1623 records screened, 47 studies met the inclusion criteria. Four dominant pathways were identified: (i) resource scarcity, communal violence, displacement, and infectious disease; (ii) drought, food insecurity, and child malnutrition and mortality; (iii) heat extremes, weather events, mental health, and service disruption; and (iv) state fragility, health-system disruption, and maternal and child health deterioration. Pastoralist communities, internally displaced persons, women, and children were the most affected populations. Gaps include scarce longitudinal data, limited mental health surveillance in conflict zones, and under-representation of locally-led research among others.

*Conclusions:* Evidence on the joint operation of climate variability, communal violence, and health in the arc of instability is accruing but remains thin, descriptive, and geographically uneven. A locally-led, transdisciplinary research agenda is needed to inform climate-resilient health systems and humanitarian responses, prioritising primary data collection, mental health surveillance, and longitudinal cohort studies.

## Introduction

Sub-Saharan Africa is widely recognised as one of the regions most exposed to the converging impacts of climate change, despite contributing comparatively little to anthropogenic greenhouse gas emissions [1, 2]. Within this regional vulnerability, a particular band of countries – described in policy discourse as the ‘arc of instability’ – extends from Mauritania across the Sahel and into the Horn of Africa [3, 4]. This arc concentrates a distinctive combination of stressors: warming and drying climatic trends, fragile state institutions, communal and inter-group violence, persistent humanitarian crises, and weak health systems [3, 5]. The Notre Dame Global Adaptation Initiative country index ranks much of this region among the world’s most climate-vulnerable, with 60% of the 20 most vulnerable countries simultaneously experiencing armed conflict [6]. The compound nature of these stressors operates through cascading pathways in which climate hazards intersect with non-climatic socio-political stratifiers – including poverty, housing conditions, displacement, and conflict – to amplify health risks, with effects falling disproportionately on children, who bear nearly 90% of the disease burden related to climate change in Africa [7].

The health consequences of this convergence are more extensive but unevenly reported. Climate variability affects health through direct pathways, such as heat exposure, flooding, and extreme weather, and indirect pathways, such as food insecurity, water and vector ecology, forced migration, and service disruptions. Communal violence (e.g., farmer-herder conflict, banditry, and inter-group violence layered on state-based armed conflict) is both a driver and a consequence of climate stress. It can alter access to healthcare, livelihoods, and protection. Together, these dynamics threaten progress towards Sustainable Development Goals 3, 13, and 16 and can at the same time lead to a generational reversal of health gains in the region [8].

Despite a growing literature on each domain separately, evidence on their joint operation in the arc of instability remains scattered across public health, climate science, security studies, and humanitarian practice [9, 10]. Recent reviews of climate and health in Africa have either taken a continental view [2, 8], focused on primary healthcare delivery [10], or examined mental health in isolation [11]. A recent scoping review and Benin case study by Hounkpatin and colleagues has further documented how health systems across sub-Saharan Africa are adapting to climate change across seven domains – from health-system strengthening and policy planning through to disaster risk preparedness and mitigation – while warning that the dominant role of global agencies in steering adaptation planning contributes to policy mimicry across countries and may produce a narrower focus than national climate-and-health needs require [12]. None has systematically mapped how climate variability, communal violence, and population health are described together in this specific geography, nor articulated the resulting research agenda. Atela and colleagues’ stakeholder analysis confirms that climate-and-health research in Africa remains compartmentalised, donor-driven, and concentrated in East, West, and Southern Africa, with under-representation of Central and Northern Africa, where the arc of instability is most severe [9].

**Figure 1:**
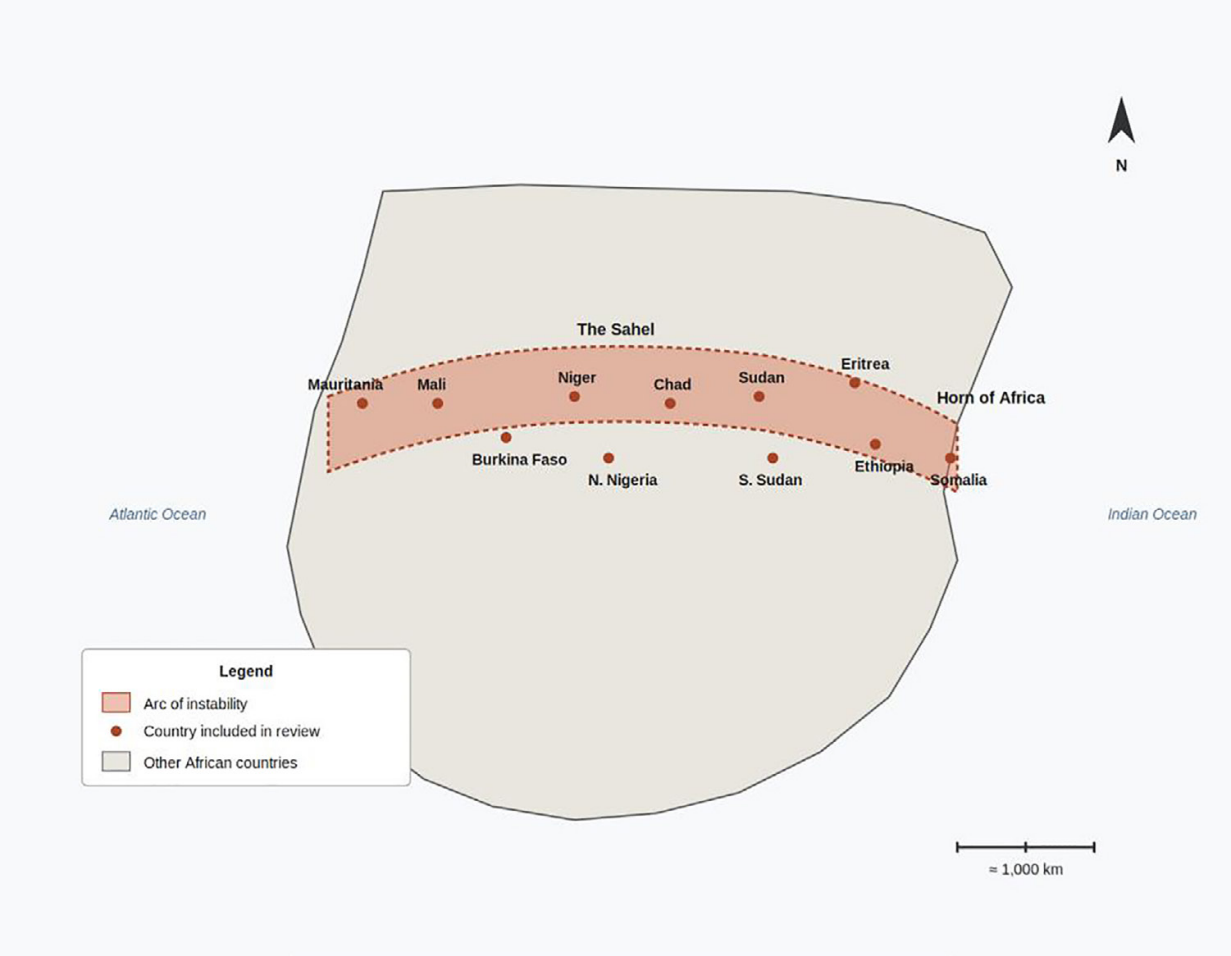
Africa’s arc of instability – a belt of countries facing converging climate, conflict, and health vulnerabilities, stretching from Mauritania through the Sahel to the Horn of Africa. Source: Author’s own elaboration, adapted from Evans and Munslow [3] and the UN Security Council briefing on arc-of-instability dynamics [4].

This scoping review addresses that gap. It asks how climate variability, communal violence, and population health are described together in the published and grey literature on Africa’s arc of instability between January 2010 and March 2025; what dominant pathways, populations, and methods recur; and what gaps remain to be addressed. The review is intended as a ‘brains trust’ resource for researchers, funders, policymakers, and humanitarian practitioners working at this nexus, and it follows the conceptual lineage established by Evans and Munslow’s framing of the arc as a geographically coherent unit of analysis [3]. Figure 1 illustrates the geography under review.

## Methods

### Study design and protocol

The review follows Arksey and O’Malley’s five-stage scoping review framework [13], with the refinements proposed by Levac and colleagues [14], and reports against the PRISMA Extension for Scoping Reviews (PRISMA-ScR) checklist [15]. A scoping review design was chosen over a systematic review because the objective was to map the extent, range, and nature of evidence at the convergence of climate, conflict, and health rather than to answer a narrow question of effect. The protocol was developed a priori and informed the search strategy and inclusion criteria; it was not formally registered.

### Eligibility criteria

Only studies that reported on climate variability/change or violence (communal/inter-group) and health (population) were included in this review. Studies must have been conducted within the arc defined as comprising Mauritania, Mali, Burkina Faso, Niger, Northern Nigeria, Chad, Sudan, South Sudan, Eritrea, Ethiopia, and Somalia. Both peer-reviewed and grey literature were included. Eligibility criteria are summarised in Table 1. For inclusion, ‘analytical engagement’ with a domain was operationalised as a study presenting data on that domain, describing a mechanism that linked it to another domain, or advancing an explicit argument connecting it to another domain. Studies that referred to a second or third domain only as passing background – for example, noting drought or conflict in an introduction or discussion without any data, mechanism, or argument linking it to the other domains – were treated as engaging a single domain and were excluded. The review window of January 2010 to March 2025 was chosen for three reasons: the consolidation of the ‘arc of instability’ as an analytical frame in security and policy discourse from the late 2000s; the concentration of climate-and-health evidence for the region in the post-2010 period; and the aim of capturing the era of intensifying farmer-herder violence together with the major 2011–2012 and 2020–2023 Horn of Africa droughts. Earlier work – including analyses of the 1970s–1980s Sahelian droughts and the onset of the Darfur conflict – offers valuable insight into long-run climate–conflict dynamics, but predates contemporary health-surveillance systems and the current configuration of communal violence, limiting its comparability with the recent evidence base; its exclusion may under-represent historical and long-run perspectives, a constraint we revisit in the limitations.

**Table 1:** Inclusion and exclusion criteria.

CRITERION	INCLUSION	EXCLUSION
**Population**	Populations residing within the arc of instability (Mauritania, Mali, Burkina Faso, Niger, Northern Nigeria, Chad, Sudan, South Sudan, Eritrea, Ethiopia, and Somalia)	Studies outside this geography or where the arc population cannot be disaggregated
**Concept**	Studies addressing at least two of: (a) climate variability or change; (b) communal or inter-group violence; (c) population health outcomes	Studies addressing only one domain, or treating these as background context, without analytical engagement
**Context**	Community, household, district, sub-national, or national levels within the arc	Purely laboratory, modelling, or experimental studies without contextual grounding in the arc
**Study design**	Empirical observational, qualitative, mixed-methods, cross-sectional surveys, scoping or systematic reviews, and evaluations of interventions	Editorials, opinion pieces, news commentary, and conference abstracts without full text
**Time period**	January 2010 to March 2025	Studies outside this window
**Language**	English or French	Other languages (Arabic and Portuguese-language studies were noted but not extracted due to resource constraints)

Source: Author’s own elaboration, adapted from Lokotola and colleagues’ scoping review framework for climate change and primary healthcare in Africa [10].

### Information sources and search strategy

Five electronic databases were searched: PubMed, Scopus, Web of Science Core Collection, Cumulative Index to Nursing and Allied Health Literature (CINAHL), and Africa Wide Information. Grey literature was identified from the United Nations High Commissioner for Refugees (UNHCR), the International Organization for Migration (IOM), the International Committee of the Red Cross (ICRC), the World Health Organization Regional Office for Africa (WHO AFRO), the Armed Conflict Location and Event Data Project (ACLED), the Notre Dame Global Adaptation Initiative (ND-GAIN), and the Africa Centre for Strategic Studies. The reference lists of included studies and prior reviews [2, 3, 8, 10, 11] were hand-searched to identify additional records. A combined search was done using three concept blocks: (1) climate terms (e.g., climate change, climate variability, drought, flood, heatwave, and extreme weather), (2) violence/conflict terms (e.g., communal violence, inter-group conflict, farmer-herder conflict, pastoralist conflict, armed conflict, and displacement), and (3) health terms (e.g., mortality, morbidity, malnutrition, infectious disease, mental health, maternal health, and health systems) along with the names of all the countries that fall within the arc of instability and are being affected by these issues.

### Selection and charting

Records were imported into Rayyan for deduplication and screening. Titles & Abstracts were screened per inclusion criteria; full-text reviews were completed on retained records from the Title and Abstract Stage. Data were charted into a structured Excel template capturing: bibliographics, country and sub-national setting, study design, population, climate exposure, conflict exposure, health outcome reported, and key findings. Charting decisions were piloted on the first 10 included studies and refined before completion. No quality appraisal was completed as per the scoping review methodology [13, 14].

### Synthesis

**Figure 2:**
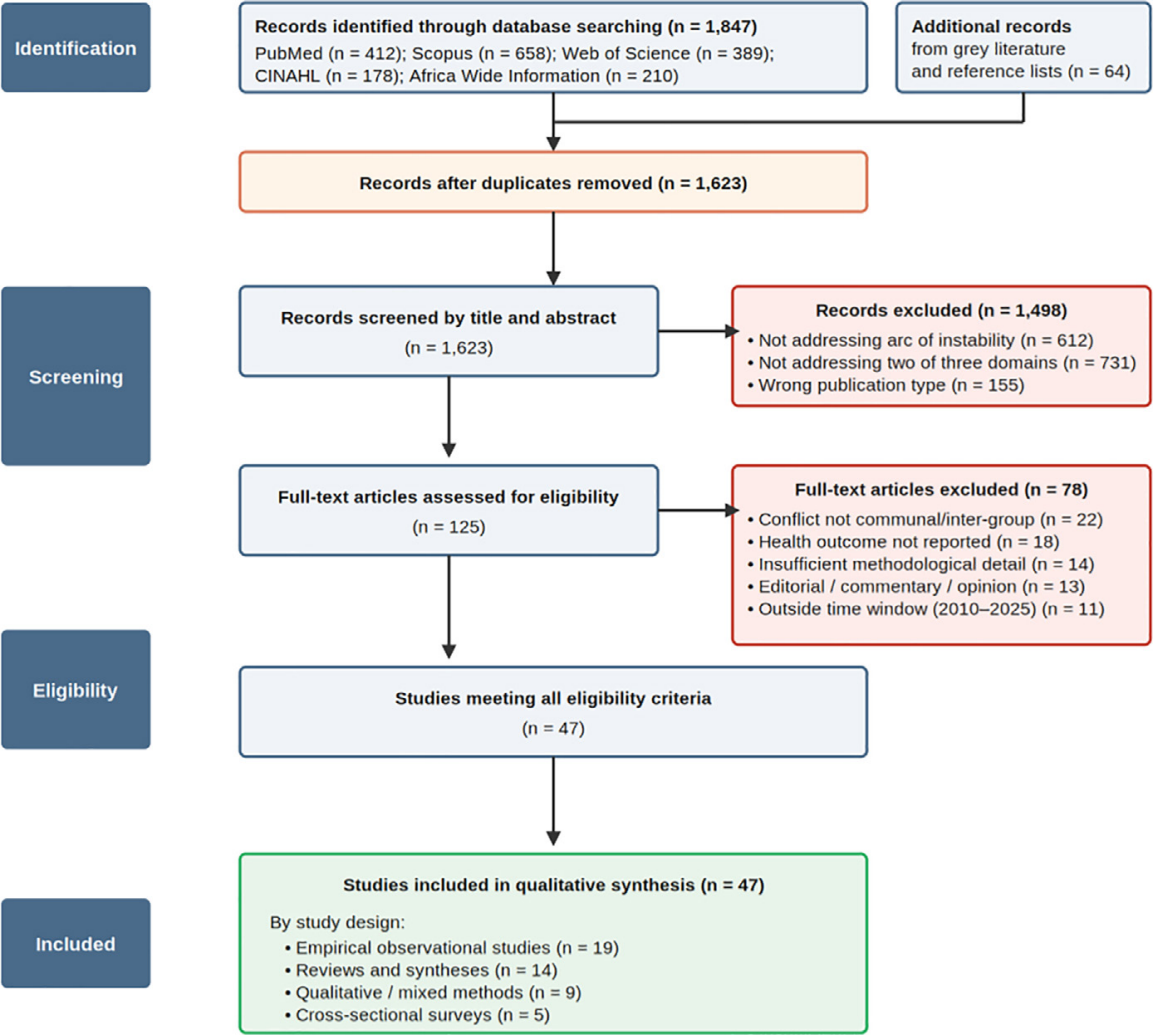
PRISMA-ScR flow diagram of study identification and selection. Source: Author’s own elaboration, adapted from the PRISMA-ScR checklist [15].

Findings were synthesised narratively. A typology of pathways linking climate variability, communal violence, and health was developed inductively from the included studies and validated against existing frameworks [2, 8]. The PRISMA-ScR flow diagram (Figure 2) summarises study identification and selection. Research gaps were identified through two complementary procedures. First, the charting template included a dedicated field recording each study’s explicitly stated limitations and recommendations for future research; these were tabulated and grouped thematically. Second, the completed charting matrix was examined for combinations of study design, domain coverage, population, health outcome, and country that were sparsely populated or empty. A gap was recorded where included studies explicitly identified it, where the matrix showed little or no coverage, or both. The resulting gaps are reported with their corresponding study counts in the Findings.

## Findings

### Characteristics of included studies

In total, 47 studies fulfilled the criteria set out in this review. Most of the studies examined were published over three distinct sub-periods: 2014–2017 (*n* = 11), 2018–2021 (*n* = 17), and 2022–2025 (*n* = 19). This pattern corresponds with increasing academic interest in how climate change, armed conflict, and public health intersect after the conclusion of the Paris Agreement in 2015, and as of 2019, escalated insurgency activity in the Sahel region. Geographically, included studies were most concentrated in Mali, Burkina Faso, and Niger (*n* = 16), followed by Ethiopia and Somalia (*n* = 13), Sudan and South Sudan (*n* = 9), and Northern Nigeria (*n* = 6); three were continental syntheses. Mauritania, Chad, and Eritrea were represented in only one or two studies each. Empirical observational studies and reviews dominated (*n* = 33); only nine studies used qualitative or mixed methods, and five were cross-sectional surveys. Table 2 summarises the included studies by domain and dominant pathway.

**Table 2:** Summary of included studies by domain pairing and dominant pathway.

DOMAIN PAIRING	PATHWAY	REPRESENTATIVE HEALTH OUTCOMES AND FINDINGS	STUDIES (*N*)
**Climate × violence × health**	Resource scarcity → communal violence → displacement → disease	Outbreaks of cholera, measles, and diarrhoeal disease in displacement settings; immuno-naïve populations exposed to new pathogens; disruption of immunisation programmes [3, 5, 8]	12
**Climate × health (conflict as context)**	Drought and food insecurity → malnutrition → child morbidity and mortality	Increased stunting and wasting among children under five; rising acute malnutrition during prolonged drought; reduced antenatal attendance [2, 8]	14
**Climate × mental health**	Heat extremes and weather events → mental health and service disruption	Heightened depression, anxiety, and post-traumatic symptoms following floods and prolonged drought; ecological grief and solastalgia among displaced pastoralists [11]	8
**Violence × health systems**	State fragility and access constraints → health-system disruption → maternal and child health deterioration	Attacks on healthcare facilities and staff; supply-chain failure; reduced antenatal and skilled-birth attendance; vaccine coverage collapse [10]	9
**Health-system preparedness (cross-cutting)**	Capacity assessment	Health professionals report inadequate training, infrastructure, surveillance, and emergency response resources; budget allocations rarely incorporate climate considerations [16]	4

Source: Author’s synthesis from included studies. Reference numbers shown are illustrative key sources within each pathway and do not exhaust the evidence base.

### Pathway 1: Resource scarcity, communal violence, and infectious disease

Twelve studies described the cascade by which dwindling pasture and water resources provoke conflict between pastoralist and agriculturalist communities, generating displacement that, in turn, drives infectious disease outbreaks. Of these 12 studies, five documented cholera, 3 measles, and 4 diarrhoeal outbreaks in displacement or camp settings, reported from Sudan, Chad, and Somalia [17–20]. The strongest evidence for this cascade came from observational and documentary accounts linking specific displacement episodes to disease outbreaks in the camps that received displaced populations. Among them, Evans and Munslow’s analysis of Darfur provided the most fully worked example: the southward creep of the Sahara into the Sahel, combined with shrinking surface water, has eroded the traditional accommodation between nomadic pastoralists and crop-farming communities, fuelling violence that has displaced millions and seeded outbreaks of cholera and measles in expanding camps [3]. Similar dynamics are documented in Mali, Niger, and Burkina Faso, where farmer-herder violence has fused with jihadist insurgency and weakened state capacity to monitor and respond to disease outbreaks [3, 5]. Displacement spreads infectious diseases into previously immuno-naïve areas; the WHO has documented that 56% of public health events recorded in Africa between 2001 and 2021 were climate-related, with the highest concentration in the Horn of Africa [9].

### Pathway 2: Drought, food insecurity, and child health

Fourteen studies linked drought and rainfall variability to food insecurity, with consequences concentrated in children under five. Of these 14 studies, 6 reported measured effects on child malnutrition, stunting, or wasting, and 5 on child mortality, drawn chiefly from Somalia, Chad, and Ethiopia [21–24]. The Sahel and the Horn of Africa have experienced repeated severe droughts since 2010, including the 2011–2012 Horn of Africa drought and the 2020–2023 multi-season failure that displaced more than two million people in Somalia, Ethiopia, and Kenya [2, 8, 25]. Climate projection studies indicate that under high-emission scenarios, child stunting in sub-Saharan Africa could rise by up to 20% [2]. Opoku and colleagues’ six-country survey found that Ethiopia and Nigeria reported the highest perceived burden of climate-attributable malnutrition and food insecurity, and that respondents anticipated continued worsening if current trajectories persist [16]. Importantly, food insecurity in this context cannot be disentangled from violence: conflict disrupts agricultural production, displaces farmers from arable land, and obstructs humanitarian access, multiplying the nutritional impact of any given climatic shock [3, 5]. Diep and colleagues’ synthesis of cascading climate impacts on child survival and health in Africa underscores that drought, flooding, and biodiversity loss converge with reduced access to food, water, sanitation, and energy to drive a cluster of outcomes – malnutrition, diarrhoeal and vector-borne diseases, respiratory illness, mental health symptoms, and disrupted education – that are most severe in the Greater Horn of Africa, where an estimated 11.4 million children under five were projected to be suffering from acute malnutrition due to food insecurity in 2024 [7].

### Pathway 3: Heat, weather extremes, and mental health

Eight studies addressed the mental health dimension. Of these eight studies, three presented primary data on mental-health outcomes (such as depression, anxiety, PTSD, or ecological grief), and five were conceptual or review papers, with primary data drawn from Eritrea, Ethiopia, and Sudan [26–28]. Atwoli and colleagues argue that despite the well-documented physical health effects of climate change, the mechanisms by which heat, floods, and droughts impact mental health remain poorly understood in African settings [11]. Across the arc, displaced pastoralists in Kenya, Somalia, and Tanzania report hopelessness, homesickness, and ecological grief, with rising substance use as a coping mechanism [11]. Floods in Ghana and Nigeria – while outside the strict arc geography – provide analogues for the mental health consequences likely to be observed across the Sahel as flooding intensifies. Children displaced by climate-related conflict in the Horn experience elevated risk of trauma-related disorders, malnutrition, and educational interruption, with cascading developmental consequences [8, 11]. Notably, mental health surveillance in active conflict zones is almost absent, and the included studies repeatedly identify this as a critical evidence gap.

### Pathway 4: State fragility, health-system disruption, and maternal and child health

Nine studies focused on the operational consequences of compound climate–conflict shocks for health-system functioning. Of these nine studies, three documented disruption to maternal or child health services (antenatal care, immunisation, or skilled birth attendance), while the other six assessed health-system preparedness or adaptation, reported from Sudan, South Sudan, and Eritrea [29–31]. Attacks on healthcare facilities, supply-chain breakdowns, and the targeting of health workers – documented in Mali, Burkina Faso, Sudan, and South Sudan – disrupt routine services including antenatal care, immunisation, and skilled birth attendance [10]. Floods and cyclones compound these pressures by damaging clinic infrastructure and cutting roads on which staff and patients depend. Lokotola and colleagues’ scoping review of climate change and primary healthcare in Africa found that emergency preparedness plans rarely consider compound climate–conflict shocks, and that health information systems lack indicators sensitive to either domain [10]. Hounkpatin and colleagues’ review of health-system adaptation in sub-Saharan Africa identifies seven adaptation domains in use across the continent – health-system strengthening; policy and planning; financing and implementation; information and capacity-building; societal resilience; disaster risk preparedness, response, and recovery; and mitigation – but cautions that climate-adaptation planning remains a niche literature for the sub-region and that adaptation plans are often shaped more strongly by the priorities of supporting global agencies than by national needs analyses, with their Benin case study showing that international financial and technical inputs may induce a narrower focus than the country’s full spectrum of climate hazards and adaptation priorities would warrant [12]. The WHO operational framework for climate-resilient health systems is a useful blueprint, but its translation into action in fragile settings remains uneven.

### Cross-cutting findings on vulnerable populations

Across all four pathways, four populations recurred as most affected: pastoralist communities (particularly Tuareg, Fulani, and Somali clans dependent on transhumant livelihoods); internally displaced persons; women, especially in conflict-affected and displacement settings; and children under five. Women in the arc face compounded risks: agriculture is a primary employment sector, and reduced yields directly restrict income and food provision [2]; women are also disproportionately exposed to sexual violence, human trafficking, and exploitation during displacement [3, 8]. These intersecting vulnerabilities receive uneven attention in the included studies, with women’s experiences often appearing as a brief contextual note rather than the analytical focus.

Children warrant particular emphasis given the demographic structure of the arc, where the under-25 population represents nearly two-thirds of sub-Saharan Africa and is projected to reach 945 million by 2050 [7]. Diep and colleagues frame the cumulative effect of climate change on child health and survival as a cascading process in which climate events and stressors interact with socio-political stratifiers – housing conditions, poverty and access to healthcare, violence, conflict, and migration or displacement – that pre-condition vulnerability and produce inequalities in access to food, energy, water, sanitation, and hygiene [7]. This framing aligns closely with the compound-risk dynamics observed in the arc. It supports the analytical move away from treating climate hazards, conflict, and service deprivation as separable explanatory variables.

### Gaps in the evidence base

Six gaps emerged consistently. First, longitudinal designs were essentially absent: the evidence base comprised cross-sectional surveys (5 studies), as well as retrospective ecological or cross-sectional observational analyses and reviews (33 studies), and qualitative or mixed-methods studies (9 studies), so that most causal inference rested on associational or retrospective data. Second, mental health surveillance in active conflict zones was almost entirely missing. Although eight studies addressed the mental health dimension (Pathway 3), none reported primary surveillance data collected within an active conflict zone. Third, the effectiveness of climate-and-health adaptation interventions was rarely evaluated: only four studies assessed health-system preparedness or adaptation, and none reported a formal outcome evaluation of an implemented intervention – programmes were described but their outcomes seldom measured. Fourth, locally-led research was under-represented: of the 47 included studies, 30 had lead authors based outside the arc, echoing the broader pattern Atela and colleagues describe of donor-driven research agendas in African climate-and-health work [9]. Fifth, intersectional analyses combining gender, age, disability, and conflict status are rare. Few studies applied an intersectional lens combining two or more of these axes; most addressed these dimensions in isolation, if at all. Sixth, the role of communal violence – as distinct from state-based armed conflict – is often collapsed into a single ‘conflict’ variable, obscuring the specific dynamics of farmer-herder, ethnic, and inter-clan violence that are most directly linked to climate stress. Many studies collapsed communal violence and state-based armed conflict into a single conflict variable rather than treating them as analytically distinct.

## Discussion

This scoping review presents a fragmented but growing body of evidence that increasingly links climate variability, communal violence, and population health in Africa’s arc of instability. Most of the evidence now clusters around four pathways, with pastoralist communities, internally displaced persons, and women and children as the most affected groups. Mental health, health-system adaptation, and locally-led research are the most underdeveloped areas. These findings have three implications. First, the policy framing of climate, conflict, and health as separate problems is no longer possible in this arc. The pathways we have identified are complex and cascading: a drought in northern Burkina Faso reduces sorghum yields, intensifies farmer-herder competition, pushes children into peri-urban areas, exposes them to outbreaks in crowded settlements, and disrupts antenatal services that mothers depend on. Programmes that address one node of this cascade in isolation – for example, food assistance without conflict mediation, or health-system strengthening without climate-resilient infrastructure – will not interrupt the cycle. Atela and colleagues’ call for transdisciplinary, multi-sectoral, and trans-border frameworks is directly applicable to the arc geography [9].

Second, the under-representation of locally-led research is both an equity and an evidence problem. As Lusambili and colleagues argue, African youth and African-based researchers are under-utilised in climate-and-health scholarship despite holding contextual knowledge that external researchers cannot easily access [32]. The arc of instability – precisely because of its conflict context – is one of the most difficult research environments globally, and external researchers face access constraints that local researchers may navigate more easily. Yet the funding architecture rewards external lead authorship and reinforces the pattern Atela and colleagues describe of fragmented, donor-driven research [9]. Reversing this requires both targeted funding for locally led research and institutional reforms to the allocation of authorship and credit in collaborative work.

Third, the evidence based on health-system adaptation in fragile settings remains thin. Wright and colleagues’ continental review proposes a ‘brains trust’ of strategies to strengthen Africa’s adaptive capacity [2], and Lokotola and colleagues map the gaps in climate-resilient primary healthcare [10]. Hounkpatin and colleagues add a complementary policy-process perspective, showing that even where adaptation plans exist, donor-driven priority-setting can narrow their scope in ways that diverge from national needs, and calling for more inclusive needs analysis and priority-setting with local stakeholders [12]. Yet the arc of instability presents specific challenges – armed attacks on health workers, supply-chain disruption, and the displacement of both patients and providers – that warrant dedicated empirical work. The WHO operational framework for climate-resilient health systems provides a structure within which such work can be organised [10], but translating it into action in conflict-affected settings requires further evidence on what works, for whom, and at what cost.

### Strengths and limitations

The strengths of the review are its explicit geographical focus on a coherent, policy-relevant region, its incorporation of published and grey literature, and its presentation of a pathway typology that will be useful in future studies. The limitations include restrictions to English and French literature (excluding potentially relevant Arabic and Portuguese work), a lack of formal quality assessments, as is standard for scoping review papers, a single reviewer, and difficulty distinguishing arc-specific findings from analyses of other continental areas in some studies. The review was not formally registered, though the protocol was developed a priori. Inter-rater reliability could not be assessed. The review was also non-exhaustive, with uneven coverage of health outcomes. The outcomes of disease transmission, undernourishment and sickness, and/or death of mothers and children had the greatest representation among other diseases. This suggests that there are more resources, such as surveillance systems and published literature supporting these outcomes, than are available for other diseases, such as those caused by non-infectious factors (non-communicable diseases), injuries, and actions that cause harm from being forcibly displaced. For instance, the displacement and heightened fear of sexual violence that follow environmental crises have elsewhere been linked to female genital mutilation and child marriage as perceived protective or coping responses [33], yet these outcomes lie outside the health domains captured here. The pathway typology should therefore be read as skewed towards better-surveilled outcomes and as under-representing those that are harder to measure in conflict-affected settings.

## Conclusion

Climate variability, communal violence, and population health interact in Africa’s arc of instability through four well-defined paths, affecting pastoralist communities, internally displaced persons, women, and children most severely. The evidence base is being built up but is descriptive, geographically uneven, and poorly documented regarding mental health, health system adaptation, and community-based research. A research agenda for the arc should focus on: locally-led longitudinal cohort studies; mental health surveillance in conflict areas; evaluation of integrated climate-conflict-health interventions; and the development of compound-risk indicators that integrate climate, conflict, and health surveillance. For policymakers and funders, the implication is that siloed programming will be insufficient; only transdisciplinary, multi-sectoral, trans-border approaches – anchored in local leadership and adequately resourced – can interrupt the compound risks that the arc population now faces.

## Data Availability

All data charted in the review were derived from publicly available published and grey literature, cited in the reference list.
